# Clinical Remission of Chronic Refractory Pelvic Symptoms in Three Men

**DOI:** 10.1100/tsw.2004.62

**Published:** 2004-06-07

**Authors:** Bradley R. Hennenfent, Antonio Espinosa Feliciano

**Affiliations:** The Prostatitis Foundation and the Manila Genitourinary Clinic, USA

**Keywords:** prostatitis, prostatodynia, prostatic massage, sexual dysfunction, infection, prostate, pelvic pain

## Abstract

We report on three American men with chronic refractory pelvic pain, urinary symptoms, and sexual dysfunction who traveled to the Philippines for treatment. In the Manila Genitourinary Clinic, the patients were treated with microbial diagnosis, antimicrobial therapy, and 19, 27, and 21 prostatic massages respectively. All three patients underwent resolution of symptoms.

## INTRODUCTION

Attempts are being made to better define the chronic pelvic and genital syndromes that involve pelvogenital pain, urinary symptoms, sexual dysfunction, and in some cases systemic symptoms such as myalgias, arthralgias, fatigue, cognitive dysfunction, night sweats, fever, or feelings of fever.

In prostatic fluid, ten or more white blood cells (WBCs) per oil immersion field (OIF, 1000 times magnification) are often used as the cutoff between prostatitis and prostatodynia. Both entities are defined as chronic when the symptoms are more than 3 months in duration.

Patients labeled chronic "non-bacterial" prostatitis or labeled chronic prostatodynia often become incurable patients who go from doctor to doctor without resolution of their symptoms.

## CASE #1

Patient #1, a 47-year-old single sexually active male, and computer software engineer from Austin, Texas, USA, appeared at the Manila Genitourinary Clinic with a chief complaint of sitting pain. The patient ranked his pain at nine out of ten in severity on a scale of 0to 10; with ten being the worst. Patient #1 traveled with a doughnut shaped pillow to sit on. The patient's history included a onetime episode of urethral discharge lasting a few days, progressive testicular pain, periodic suprapubic pain, groin tenderness, slight constriction of urinary flow, intermittent pain during masturbation, pain after ejaculation, less forceful ejaculation, and decreased semen volume. The patient's symptoms were of 7 months duration at the time of presentation.

Patient #1 saw several physicians prior to coming to Manila. At a sexually transmitted disease clinic, Human Immunodeficiency Virus (HIV), syphilis, Neisseria gonorrhea, Chlamydia, and urethral swab for culture were all negative.

The first urologist seen by patient #1 noted a urinalysis (UA) that was "strongly positive" for a urinary tract infection (UTI). Microscopic examination of the expressed prostatic secretions (EPS) revealed 50-60 WBCs and 10-15 red blood cells (RBCs). Diagnoses of UTI and prostatitis were given. The patient was treated with ciprofloxacin bid for 6 weeks and then was switched to ofloxacin for several weeks without resolution of symptoms.

The second urologist seen by patient #1 recorded a tender prostate with "loaded" WBCs in the EPS. Chlamydia culture of the EPS was negative. Routine cultures of the EPS were negative. Semen cultures were negative. Prostatitis was diagnosed. Azithromycin for one dose, and doxycycline for 7 days were prescribed. The patient's symptoms did not resolve. Cardura was tried without significant relief. Ibuprofen was also not significantly effective.

Patient #1's last consultation was at the University of Washington (UW). The University of Washington at that time was the only American university receiving funding from the National Institutes of Health for prostatitis research, and has been pledged 3.2 million dollars in private funding by the Paul Allen Foundation for prostatitis research.

Patient #1's results at UW revealed 0 urethral WBCs and a urethral culture for Neisseria gonococcus was negative. The Meares and Stamey 4 Glass revealed voiding bladder one (VB1): 0 WBC and 1420 colony/ml Staphylococcus coagulase negative; VB2: 0 WBC and 110 colony/ml Staphylococcus coagulase negative; EPS 1-5 WBC/HPF and 200 colony/ml Staphylococcus coagulase negative; and VB3: less than 1 WBC/HPF and 500 colony/ml Staphylococcus coagulase negative. EPS cultures for Neisseria gonorrhea, Chlamydia, Mycoplasma, Ureaplasma, and Trichomonas were all negative. Rectal color Doppler ultrasound showed increased blood flow to the prostate. The University of Washington was doing a study where they biopsied prostates and used PCR primers to detect microorganisms, but the patient #1 declined to participate in the invasive study. No etiology of illness was discovered, and no treatment was offered the patient.

In the Manila Genitourinary Clinic, the patient was a normal appearing circumcised male. A direct immunofluorescence (DIF) test on a urethral smear for Chlamydia was negative. Urethral Gram's stain showed 4 to 10 WBCs, yeast, and Gram-positive cocci. Digital rectal examination determined the prostate to be normal in size. The examining physician felt a popping sensation during prostatic massage, which to him indicated the release of obstructed prostatic ducts. Two drops of cloudy prostatic fluid with precipitates were expressed. EPS cultures were done for aerobic and anaerobic bacteria. The EPS was also cultured for Mycoplasma, Ureaplasma, and Trichomonas. The only organism that grew was Staphylococcus epidermidis. A urinalysis after prostatic massage was performed which revealed 0 to6 WBCs.

In the Manila Genitourinary Clinic, prostatic fluid WBC counts are done by collecting the first drop of EPS and examining twenty fields of the Gram's stain under oil immersion field (1000 power). The high and the low count in specimens are recorded. Patient #1 had 19 massages with collection of his EPS and Gram's stain. The first EPS WBC count in patient #1 was 10 to 25. The changes in patient #1's EPS WBC counts over 19 massages are shown in figure1.

At the first visit, patient #1 was given fluconazole 150 mg as a single dose for yeast seen in the Gram's stain of a urethral swab. Patient #1 was also given metronidazole 2 grams p.o. empirically as a single dose because of the high white blood cell count in the EPS.

At the second massage ofloxacin 400 mg bid and doxycycline 100 mg bid were started based on the sensitivities of the Staphylococcus epidermidis to antibiotics. Urethral inflammation disappeared after the second visit.

At the third visit, yeast was again seen in the EPS and fluconazole 150 mg as a single dose was repeated. The ofloxacin and doxycycline were continued.

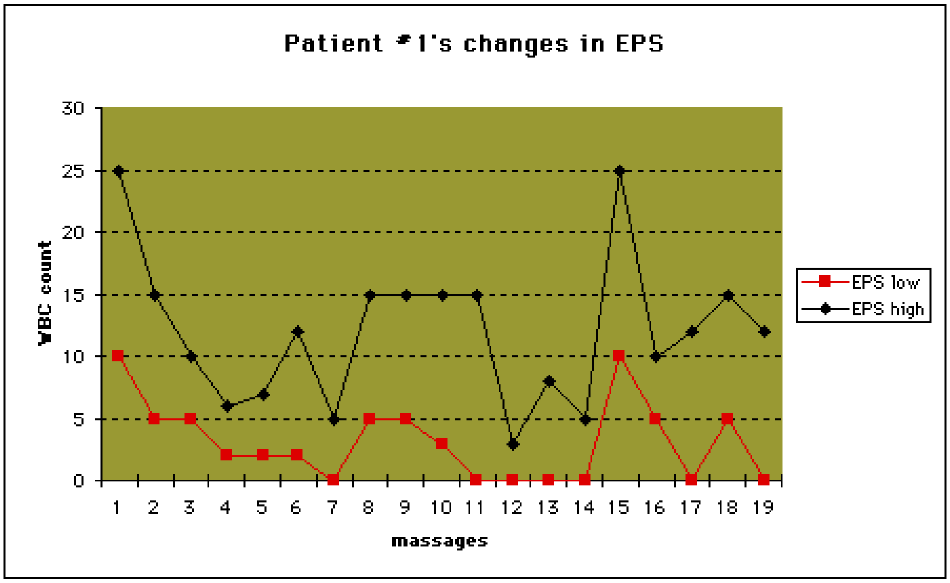

**FIGURE 1**. Changes in the WBC counts of patient #1 over 19 prostatic massages.

At the ninth visit, metronidazole 2 grams p.o. times one dose was again given because of the high number of WBCs in the EPS. At the 11th massage, doxycycline was stopped because the patient was having difficulty tolerating the medication. At the 14th massage, minocycline was added because Staphylococcus Epidermidis grew again and was sensitive. At the 17th massage, antibiotics were stopped. Itraconazole 200 mg bid times for one day, then 100 mg once a day for two days was given empirically to prevent yeast overgrowth. Patient #1's Gram's stains and cultures are summarized in table1.

Using the 0 to 10 symptom score, with zero being no symptoms and ten being the worst possible symptoms, patient #1 started out at 9 on his symptom score. The symptoms dropped to 0 at the ninth massage, and consistently stayed at 0 after the 13th massage (figure2). The last three cultures for Staphylococcus epidermidis were all negative.

During treatment, patient #1 was prescribed a total of 5 different antimicrobials. These are summarized in table2.

## CASE #2

Patient #2, a forty-year-old single sexually active male, a medical equipment salesman from Philadelphia, Pennsylvania, USA presented to the Manila GU Clinic with a chief complaint of intermittent perineal pain for one and one-half years. He also complained of left testicular pain, rectal pain, pain in the lower back, pain in the penis, pain during urination, pain in the upper thighs, the bladder not feeling completely empty after urination, the need to urinate again less than 2 hours after urinating, stopping and starting during urination, difficulty postponing urination, weak urinary stream, having to push or strain to urinate, getting up one or more times a night to urinate, difficulty ejaculating, difficulty maintaining an erection, night sweats, and reported intermittent low-grade fever for several months, myalgias, and arthralgias.

Patient #2 saw several physicians prior to arrival in the Philippines. At an STD clinic, Neisseria gonorrhea, syphilis, Chlamydia, and HIV were negative, as was a urethral swab for culture. Patient #2 went to multiple other physicians including three urologists. Repeated urinalyses were negative. One urologist at Temple University attempted a Meares and Stamey localization procedure but no EPS was obtained. Patient #2 was treated with many antibiotics including doxycycline, sulfamethoxazole, ofloxacin, amoxicillin/clavulanate, fluconazole, and metronidazole. He was given various different diagnoses including prostatitis, urethritis, and epididymitis. Ibuprofen was taken without effect.

**TABLE 1**
**Results of positive Gram stains and cultures for patient #1.**

| Patient #1 massages | Results of Gram's Stains | Results of cultures |
|---|---|---|
| 1 | Yeast in urethra, Gram-positive cocci in urethra | Staphylococcus epidermis |
| 2 | | |
| 3 | Yeast in EPS | |
| 4 | Gram-negative diplococcus (extracellular) in EPS | |
| 5 | Gram-negative diplococcus (extracellular) in EPS | |
| 6 | Gram-positive cocci in EPS | |
| 7 | | |
| 8 | | |
| 9 | | |
| 10 | | |
| 11 | | |
| 12 | | |
| 13 | | Staphylococcus epidermidis |
| 14 | | |
| 15 | | Staphylococcus epidermidis (possible) |
| 16 | | |
| 17 | | |
| 18 | | |
| 19 | | |

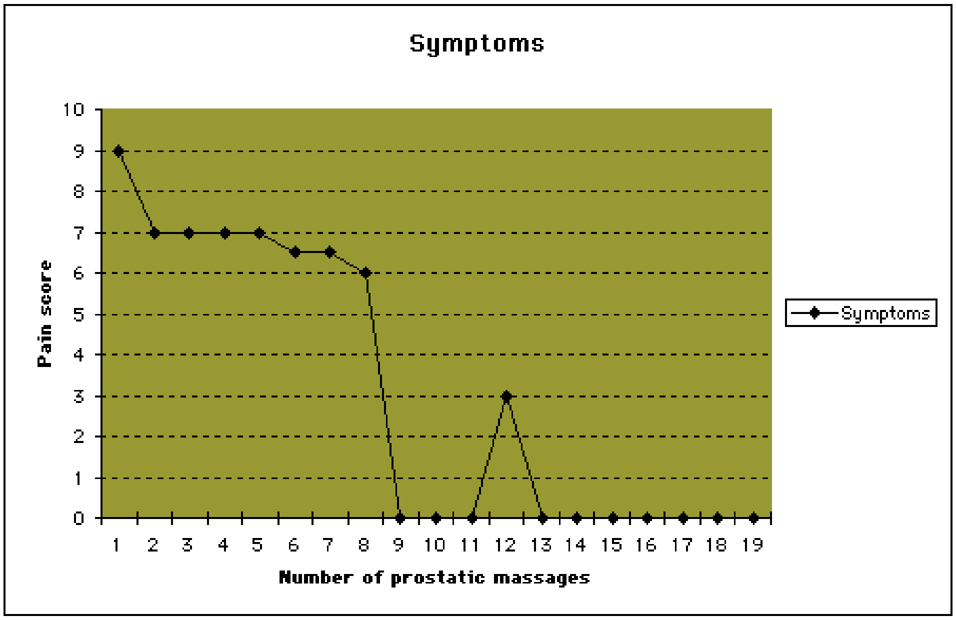

**FIGURE 2.** The dropping pain score of patient #1 over 19 prostatic massages.

**TABLE 2**
**List of all antimicrobial agents prescribed for patient #1.**

| Antimicobial agents prescribed patient #1 |
|---|
| fluconazole |
| metronidazole |
| ofloxacin |
| doxycycline |
| itraconazole |

During the first visit at the Manila Genitourinary Clinic, the physical examination revealed normal circumcised genitalia. The Gram's stain of the urethra showed 0 to 2 WBCs. The prostate was enlarged and boggy. A Gram's stain of the EPS revealed 0 to 4 WBCs. A UA after prostatic massage was normal. Cultures for aerobes, anaerobes, Mycoplasma, Ureaplasma, and trichomonas were done, and were negative. A direct immunofluorescence test for Chlamydia of the urethra was also done. Ofloxacin 400 mg bid was started empirically.

The patients Chlamydia DIF was known to be positive at the second visit, and minocycline 100 mg bid was added. At the third massage itraconazole 100 mg 2 caps bid times 1 day was given for yeast in the urethra and the EPS. At the fourth visit, ofloxacin was stopped because of CNS side effects. Minocycline was continued until the 12$^{th}$ massage.

At the 13$^{th}$ visit, all cultures were repeated. Chlamydia DIF was negative. Itraconazole 100mg 2 caps bid times one day, and then 100 mg a day was started empirically because of the course of antibiotics. Because of RBCs seen in the EPS by the 14$^{th}$ massage (usually associated with Staphylococcus epidermidis or yeast in the clinic's experience), a switch in antifungals to ketoconazole 400 mg per day was made.

At the 15$^{th}$ visit, Staphylococcus epidermidis had grown. Amoxicillin/clavulanate 625 mg tid was started. At the 22$^{nd}$massage, ketoconazole 400 mg a day was started.

Cultures at the 23$^{rd}$ massage were negative. The Amoxicillin/clavulanate was stopped. Fungus was seen in the EPS of the 23$^{rd}$, 24$^{th}$, and 25$^{th}$ massages. Antifungal therapy was switched to itraconazole 200 mg bid x one day, then 100 mg q day x 19 days. No Staphylococcus or fungus was present at the 26$^{th}$ massage or the 27$^{th}$ (last) massage.

A summary of patient#2's Gram's stains and cultures are in table3. The EPS WBC counts over 27 massages in Figure3. Patient #3 received a total of 5 different antimicrobials (table4).

By the end of massage therapy, Patient #2's prostate was decompressed by the massages and the consistency of his prostate had improved. His symptom score started at 10 out of 10 in severity and dropped to 0 (no symptoms) by the end of the treatment protocol (figure4).

## CASE #3

Patient #3, a 40 year-old divorced sexually active criminal investigator, presented to the Manila Genitourinary Clinic with suprapubic pain and lower back pain of 15 months duration. He also complained of pre-mature ejaculation, pain after ejaculation, intermittent impotence, decreased quality of erections, myalgia, arthralgias, fatigue, and feelings of fever.

The patient's first entry into the health care system had been to the emergency department New York City after sudden onset of pelvic pain and feeling feverish. He was given muscle relaxants, which did not relieve his symptoms.

**TABLE 3**
**Results of Gram stains and cultures for patient #2.**

| Patient #2 massages | Gram's stain results | Culture results |
|---|---|---|
| 1 | | Neisseria species and Staphylococcus species from EPS, Chlamydia DIF positive from urethral swab |
| 2 | Yeast in urethra, Gram-negative coccobacilli in EPS, Yeast in EPS | |
| 3 | Yeast in urethra, Gram-negative coccobacilli in EPS, Gram-negative diplococcus (extracellular) in EPS | |
| 4 | Yeast in urethra, yeast in EPS | |
| 5 | Yeast in urethra, Gram-negative diplococcus (extracellular) in EPS | |
| 6 | Yeast in urethra, Gram-negative diplococcus (extracellular) in EPS, Gram-positive cocci | |
| 7 | Yeast in EPS | |
| 8 | | |
| 9 | | |
| 10 | | |
| 11 | Gram-negative diplococcus (extracellular) in EPS, Gram-positive cocci in EPS | |
| 12 | | |
| 13 | | Staphylococcus epidermidis |
| 14 | | |
| 15 | | |
| 16 | | |
| 17 | | |
| 18 | Gram-positive cocci in EPS | |
| 19 | | |
| 20 | | |
| 21 | | |
| 22 | Gram-positive cocci in EPS | |
| 23 | Yeast in EPS | |
| 24 | Yeast in EPS | |
| 25 | Yeast in EPS | |
| 26 | Gram-positive cocci in EPS | |
| 27 | | |

Patient #3's local urologist noted prostate tenderness and microscopic hematuria. A cystoscopic examination revealed redness of the prostatic urethra. A ten-day course of ofloxacin was given with relief of symptoms. Symptoms returned 2 days after coming off the antibiotic. Ofloxacin for 6 weeks was given, with some relief, but again symptoms returned after the antibiotic was stopped.

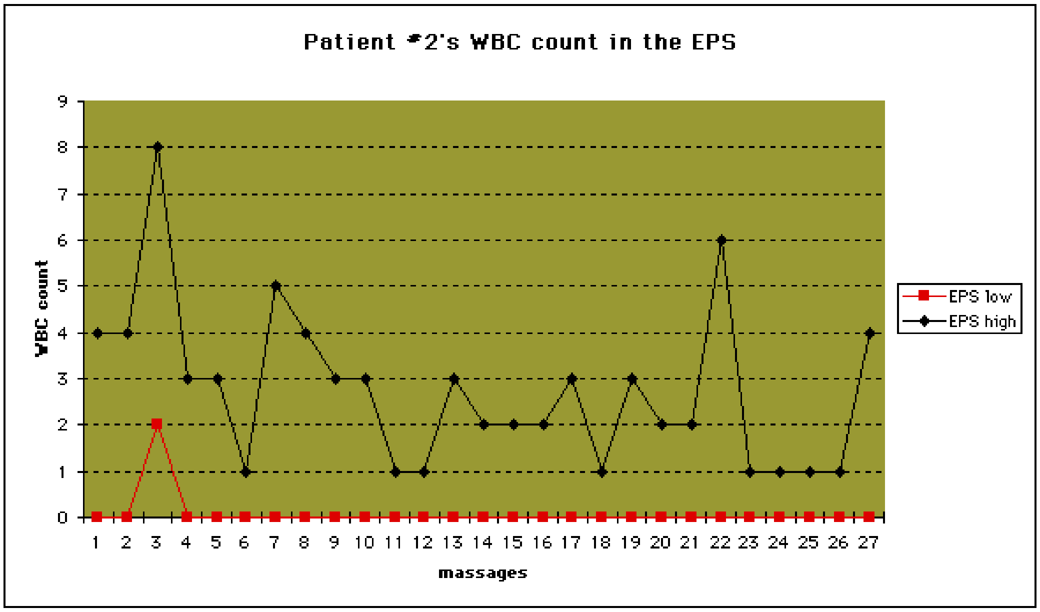

**FIGURE 3.** Changes in the WBC counts of patient #2 over 27 prostatic massages.

## TABLE 4
## List of all antimicrobial agents prescribed for patient #2.

| Antimicobial agents prescribed patient #2 |
|---|
| ofloxacin |
| minocycline |
| itraconazole |
| amoxacillin/clavulanate |
| ketaconazole |

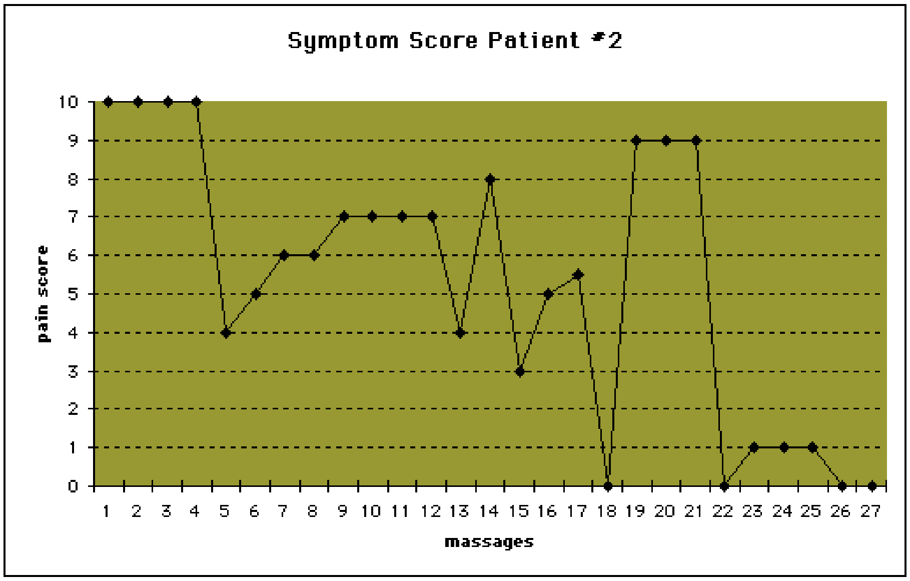

**FIGURE 4.** The dropping pain score of patient #2 over 27 prostatic massages.

At Mount Sinai Medical Center in New York, a urologist performed an ultrasound and did another cystoscopy. Again, erythema of the prostatic urethra was noted. Doxycycline for 4 weeks and a non-steroidal antiinflammatory was given with intermittent relief of symptoms. Ciprofloxacin was prescribed but symptoms continued. The patient tried various herbal therapies without relief. The patient visited a urologist at Cornell Medical Center, where he was told he would have to live with his symptoms.

An obstetrician-gynecologist gave patient #3 intravenous gentamicin and clindamycin with a decrease in symptoms. Symptoms returned after the medications were stopped.

Another physician prescribed diazepam, which gave transient relief of symptoms. The nonsteroidal antiinflammatory oxaprozin (Daypro) also had temporary benefit.

At the Manila GU Clinic, the physical exam revealed normal circumcized genitalia. The urethral WBC count was 0-1. A Gram's stain of the urethra showed Gram-negative coccobacilli and Gram-positive cocci. The EPS WBC was 0-2. Changes in this patient's EPS WBC count as therapy continued are shown (figure5). At the second massage, Gram-negative diplococci (extracellular) were seen in the EPS.

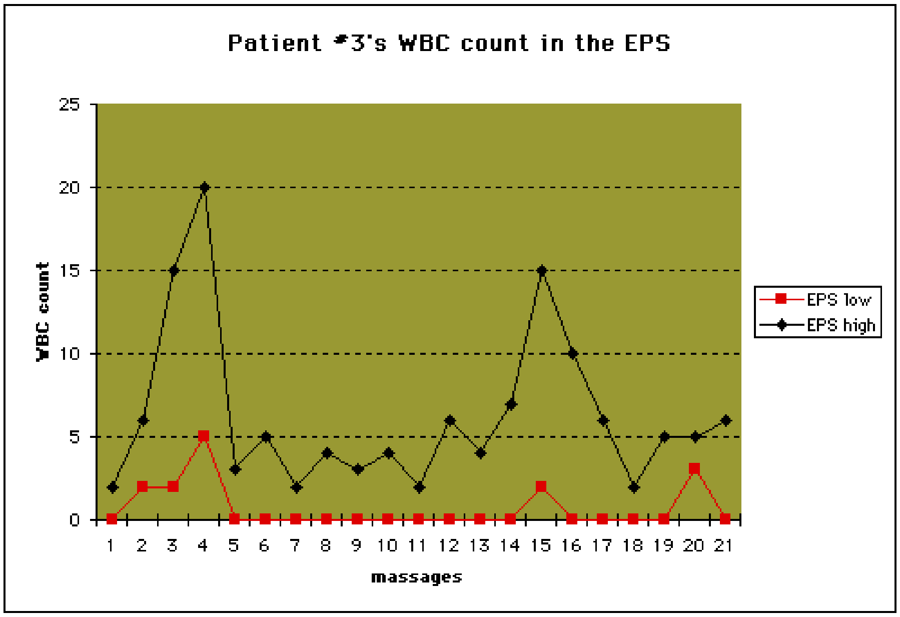

**FIGURE 5**. Changes in the WBC counts of patient #3 over 21 prostatic massages.

At the third massage Staphylococcus aureus had grown and minocycline 100 mg bid was started. Since multiple bacteria were seen on the Gram's stain of the fourth massage's EPS, ciprofloxacin was added.

The 4th massage revealed 5-20 WBCs in the EPS, and metronidazole, 2 grams p.o., as a single dose was given empirically.

Ciprofloxacin was stopped at the 13$^{th}$ massage and minocycline was stopped at he 14$^{th}$ massage. Itraconazole was started empirically at the 13$^{th}$ massage. A summary of patient #3's Gram stains and cultures are shown in table5.

Patient's #3's symptom score went from10 (worst possible) to 0.5 (on the 0 to 10 symptom score) by the end of treatment (figure6). While in Manila, patient #3 received a total of 4 different antimicrobials (table6). For financial reasons, patient #3 returned to the USA where he sought out a physician willing to continue to do massages and give antimicrobial therapy. He received both massages and antibiotics for several more weeks. Finally, even the last 0.5 of his symptoms resolved. He remains completely asymptomatic today.

**TABLE 5**
**Results of Gram stains and cultures for patient #3.**

| Patient #3 massages | Results of Gram's stain | Results of Culture |
|---|---|---|
| 1 | Gram-negative coccobacilli in urethra, Gram-positive cocci in urethra | Staphylococcus aureus |
| 2 | Gram-negative diplococci (extracellular) in EPS | |
| 3 | | |
| 4 | Gram-negative cocci in urethra, Gram-negative diplococci (extracellular) in urethra, Gram-negative bacilli in urethra, Gram-positive cocci in urethra | |
| 5 | | |
| 6 | | |
| 7 | | |
| 8 | | |
| 9 | | |
| 10 | | |
| 11 | | Staphylococcus epidermidis |
| 12 | | |
| 13 | | |
| 14 | | |
| 15 | | |
| 16 | | |
| 17 | | |
| 18 | | |
| 19 | | |
| 20 | | |
| 21 | | |

## DISCUSSION

This paper presents the case reports of three patients with symptomatic cures of chronic refractory pelvic symptoms, and describes their diagnostic and treatment protocols in detail. That these patients' symptoms are consistent with what is commonly diagnosed as nonbacterial prostatitis or prostatodynia is clear by comparison to recent studies,[1,2] and from the results of their laboratory studies.

This study is too small to make any valid conclusions about correlation between white blood cell counts and symptom scores. Nevertheless, correlation coefficients were calculated. In 5 of 6 instances a weak correlation was found between the WBC counts and the symptom scores (table 7). It is hoped that a future study with greater sample size will determine if a true relationship exists between WBCs in the EPS and symptom scores.

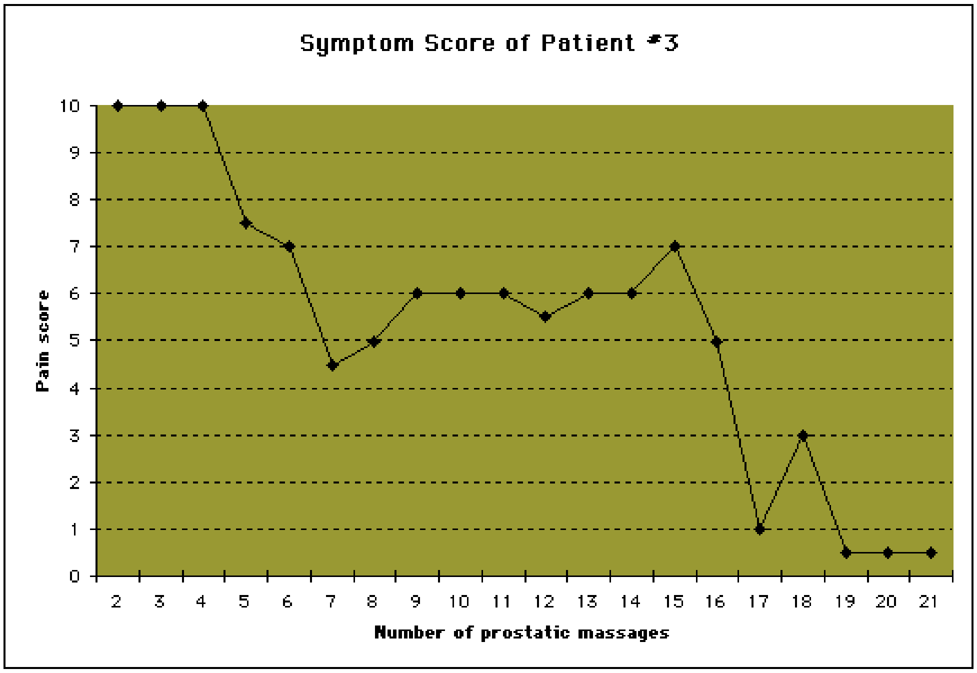

**FIGURE 6**. The dropping pain score of patient #3 over 21 prostatic massages.

**TABLE 6**
**List of all antimicrobial agents prescribed for patient #3.**

| Antimicobial agents prescribed patient #3 |
|---|
| minocycline |
| ciprofloxacin |
| metronidazole |
| itraconazole |

**TABLE 7**
**The correlation coefficients between WBC counts and dropping pain scores in the three patients.**

| | Low WBC count correlation | High WBC count correlation |
|---|---|---|
| **Patient #1** | 0.23338 | -0.03583 |
| **Patient #2** | 0.26238 | 0.29925 |
| **Patient #3** | 0.27448 | 0.26368 |

Prostatitis is separated into four categories: acute bacterial, chronic bacterial, nonbacterial, and prostatodynia by cultures and the WBC count in the EPS. Ten or more white blood cells per oil immersion field (OIF 1000 x magnification) is arbitrarily used as the division between prostatodynia and prostatitis.[5,6,7,8] The authors have shown in prior work that a one-time collection of EPS is misleading for making a diagnosis and does not allow for the most purulent specimens to be collected for Gram's stain and culture.[9] Urologist Gordon Pilmer reached the same conclusion in 1962, when he wrote: "Often six or eight 'provocative' massages will be necessary before pus can be found in the secretion."[10]

The Meares and Stamey localization test has been considered the gold standard for diagnosing the prostatitis syndromes since 1968 when it was published based on only 6 patients.[11] Using this method approximately 90% of patients with chronic pelvic symptoms are labeled chronic nonbacterial prostatitis or prostatodynia.[12,13]

In early 1996, reports started appearing on the Internet Usenet newsgroup, sci.med.prostate.prostatitis from men claiming to have been cured of chronic nonbacterial prostatitis or prostatodynia by a treatment protocol in the Philippines. These reports were noteworthy because some of these men had been to multiple urologists and had undergone extensive workups.

The Manila Genitourinary (GU) Clinic's protocol consists of thrice-weekly (or more frequent) prostatic massage, microbial testing and antimicrobial therapy. The protocol essentially combines two "gold standard" therapies for prostatitis: repetitive prostate massage and antibiotics. Pathologists recognize obstruction as a component of prostatitis;[14] however, the concept of prostatic massage was largely dropped as a therapy after the introduction of antibiotics in the 1970's without proper clinical studies ever being done. The Manila treatment protocol is based on the principle that to cure infection, obstruction must also be relieved.[15]

It is the intent of the physicians in the Manila GU Clinic to completely drain the entire prostate of secretions by thrice-weekly, or more frequent, prostatic massage. Massage is done in the direction in which the glands drain. The prostate is not pressed straight down upon. Pressure is applied from the lateral margins obliquely towards the midline grove down one side of the prostate and then the other, from the superior pole of the prostate to the inferior apex to drain the peripheral zone. The central zone glands drain more directly straight down from the back of the prostate to the verumontanum in a cephalad to caudal direction. The massages are meticulous, with every aspect of the reachable prostate being drained. Increasing pressure is applied during the thrice-weekly massages as tolerated by the patients.

The anatomic feature of the prostate that makes drainage of the gland possible by DRE is that all glandular tissue is posterior to the urethra. The anterior prostate is fibromuscular tissue not glandular. The Manila GU Clinic physicians do not believe that the prostate itself can be damaged if it is massaged in an anatomically correct manner. Theoretical concerns with thrice-weekly prostatic massage are bradycardia, syncope,[16] exacerbation of heart disease, anal fissure, or irritation of hemorrhoids. No harmful side effects were seen in these three patients.

Using the above technique of prostatic massage one of the authors (Feliciano) obtained EPS in 100% of attempts in these three patients.

Despite Gram-positive infections of the prostate documented by the Meares and Stamey technique, and electron microscopic evidence of Gram-positive organisms in prostate tissue biopsies, the role of Gram-positive bacteria as pathogens in prostatitis remains controversial.[17,18,19,20] Since assuming Gram-positives are contaminants puts the majority of prostatitis patients into the incurable category of nonbacterial prostatitis or prostatodynia,[21,22,23] the Manila Genitourinary Clinic assumes that Gram-positive bacteria are either primary or secondary bacterial pathogens, and assumes that the prostatic fluid should be sterile as seen on Gram's stain and by culture. This approach is opposite the conventional wisdom in North America and Europe, however; it is a recognized surgical principle that indigenous microbial flora can cause disease when local host barriers break down.[24]

There has been a movement to remove prostatodynia from the classification of prostatitis syndromes.[25] In light of the paper by Tulane researchers demonstrating bacteria (mostly coagulase negative staphylococci) in prostatodynia patients that was not present in controls,[26] prostatodynia deserves a second look using the technique of repetitive prostatic massage.

A criticism has been made, suggesting that repetitive prostate massage causes white blood cells to appear in the prostate fluid. The authors have done a Medline search and a manual literature search and have not found a documented case in the medical literature suggesting that massage of any gland in the human body causes pus to occur in its secretions. Nonetheless, further study is warranted.

The authors do not discount the idea that some as yet unidentified bacteria may be responsible for prostatitis in many cases. In lieu of the use of DNA technology to conquer other inflammatory diseases[27] once of unknown etiology such as Cat-scratch disease, bacillary angiomatosis, Whipple's disease and ulcer disease, the authors suggest using DNA technology on the most purulent prostatic fluid secretions to see if unculturable organisms are involved. Krieger, et al., found bacterial 16s rRNA in 77% of patients with "nonbacterial" prostatitis from perineal prostate biopsy specimens in which skin contamination was controlled. In addition, another 8% of the patients had Mycoplasma genitalium, Chlamydia trachomatis, or Trichomonas Vaginalis.

These 3 patients were cultured for anaerobes, and even though the cultures were negative the patients were still treated empirically with metronidazole.[29] Anaerobes have been implicated in prostate disease, even causing prostatic abscess.[30,31,32,33,34,35] The Manila Genitourinary Clinic treats anaerobes empirically on the assumption that all these bacteria cannot be cultured or are too expensive to culture with current culture methods.[36] This is similar to how pelvic inflammatory disease in women is treated.

Antifungal agents were prescribed on the basis of budding yeast seen in the Gram's stains of the urethral smears or EPS, or empirically after courses of antibiotics.

The authors believe that new technology must be brought to bear against the chronic urogenital syndromes. DNA technology should be used to map out the normal flora of the urogenital system using the localization technique of Meares and Stamey, while also controlling for the organisms on the skin of the penis. EPS should be examined using all culture methods and all DNA techniques in order to delineate completely all pathogens. It is important to note that the Meares and Stamey localization technique either was not done, or did not play a role in the cure, of any of these patients.

Finally, and independent study appears to support the concept of repetitive prostatic massage combined with antimicrobial therapy in patients with chronic pelvic symptoms. The study's abstract by urologist Daniel Shoskes, entitled, *Evaluation of Combined Antibiotic Therapy and Regular Prostatic Massage for Chronic Prostatitis* is presently on the World Wide Web at http://www.ben2.ucla.edu/~dshoskes/abstracts.html. Dr. Shoskes treated 28 patients who had been seen by an average of 4.5 physicians prior to treatment without clinical cure. Forty-three percent of the patients in the study; however, underwent complete resolution of symptoms, and 36% had some improvement in symptoms with repetitive prostatic massage and antimicrobials.[37]

## CONCLUSION

Three continuously symptomatic patients with chronic refractory pelvic pain syndromes are presented who had been diagnosed as prostatitis after being seen at reputable institutions. The patients were entered into the Manila Genitourinary Clinic protocol of thrice-weekly prostatic massage, antimicrobial diagnosis, and antimicrobial therapy. All three patients underwent resolution of their symptoms as determined by a global symptom score, and remain free of symptoms after treatment at 29 months, 26 months, and 15 months respectively.

## ACKNOWLEDGMENTS

The consultant statisticians for this paper were Jesus N. Sarol, Jr., Msc, and Raphael Isingo, from the University of the Philippines College of Public Health. Thanks to patient #1, patient #2, and patient#3 for opening their medical records to the authors, for proofreading the article, and for providing letters to accompany this article verifying its accuracy and authenticity.
